# Obstetric Intensive Care Unit Admission: A Six Year Cohort Study

**DOI:** 10.1186/2197-425X-3-S1-A525

**Published:** 2015-10-01

**Authors:** A Ruimy, B Calon, A Guillaume, C Meyer, M Barone, A Launoy, B Langer, P Diemunsch

**Affiliations:** Hôpitaux Universitaires de Strasbourg, Anesthésie Réanimation, Strasbourg, France; Hôpitaux Universitaires de Strasbourg, Gynécologie Obstétrique, Strasbourg, France

## Introduction

One in a thousand pregnancies will be complicated by serious maternal morbidity warranting intensive care unit (ICU) admission before, during or after delivery. Between 2007 and 2009 maternal mortality in France was one in ten thousand, about half of them are considered preventable.

## Objectives

In order to assess and improve avoidable severe maternal morbidity (SMM), whether lethal or not, a prospective analysis was undertaken in a tertiary care obstetric ICU (TCO-ICU) over a six year period.

## Methods

Between January 2007 and December 2012 data were collected on all obstetric and post-partum admissions in the Strasbourg (France) TCO-ICU.

## Results

Among the 305 TCO-ICU admissions, 2 patients presented with avoidable (iatrogenic) non-lethal morbidity and 8 patients died, all of them from non-avoidable causes. No deaths occurred in the 2 main groups, hypertensive disorders of pregnancy and major obstetric hemorrhage.

## Conclusions

Optimal cooperation between our tertiary facilities and non-teaching hospitals and clinics as well as the prehospital transport systems allows significant limitations in avoidable obstetric maternal morbidity and mortality. Cerebral hemorrhage and amniotic fluid embolism carry the heaviest toll in non-avoidable deaths.

**Table 1:** *Iindications for ICU admission.*

indication for ICU admission	number of patients	%	number of deaths
hypertensive disorder	195	64	0
Hemorrhage	51	16.7	0
infectious disease	11	3.6	1
amniotic fluid embolism	9	3	2
hepatic disorder	6	2	0
cerebral disease	5	1.6	3
cardiac disease	4	1.3	0
thromboembolism	3	1	0
miscellaneous disorders	16	5.4	2
